# Investigation on the Influence of Fiber Bundle Undulating Architecture on Tensile Behavior of Filament Wound Composite Laminates

**DOI:** 10.3390/ma16103697

**Published:** 2023-05-12

**Authors:** Hao Liu, Haixiao Hu, Dongfeng Cao, Yundong Ji, Xiangjiang Wang, Hongda Chen, Shuxin Li

**Affiliations:** 1 Hubei Key Laboratory of Theory and Application of Advanced Materials Mechanics, Wuhan University of Technology, Wuhan 430070, China; 2 Foshan Xianhu Laboratory of the Advanced Energy Science and Technology Guangdong Laboratory, Foshan 528000, China; 3 Institute of Advanced Materials and Manufacturing Technology, Wuhan University of Technology, Wuhan 430070, China; 4 State Key Laboratory of Advanced Technology for Materials Synthesis and Processing, Wuhan University of Technology, Wuhan 430070, China

**Keywords:** filament wound composites, fiber bundle undulating architecture, digital image correlation, finite element model

## Abstract

In filament wound composites, fiber bundles cross each other and form an undulating architecture, which may significantly affect the mechanical behavior of composites. In this study, the tensile mechanical behavior of filament wound laminates was studied experimentally and numerically, and the influences of the bundle thickness and winding angle on the mechanical behavior of the filament wound plates were also explored. In the experiments, tensile tests were carried out on filament wound plates and laminated plates. It was found that, compared to laminated plates, filament wound plates had lower stiffness, greater failure displacement, similar failure loads, and more obvious strain concentration areas. In numerical analysis, mesoscale finite element models, which take into account the fiber bundles’ undulating morphology, were created. The numerical predictions correlated well with the experimental ones. Further numerical studies have shown that the stiffness reduction coefficient of filament wound plates with a winding angle of ±55° decreased from 0.78 to 0.74 as the bundle thickness increased from 0.4 mm to 0.8 mm. The stiffness reduction coefficients of filament wound plates with wound angles of ±15°, ±25°, and ±45° were 0.86, 0.83, and 0.8, respectively.

## 1. Introduction

Filament wound composites (FWCs) are high-performance fiber-wrapped composite structures that have several advantages, such as ease of manufacturing and shaping, a high degree of automation, and a design which is simple to implement. FWCs have been widely used in the fields of aerospace, national defense, transportation, and many others. Some examples include high-pressure hydrogen storage tanks, solid missile launch tubes, aircraft radomes, S-shaped intakes, helicopter rotors, and other complex structures [1,2,3,4].

In FWC structures, fiber bundles cross and undulate with each other, which is different from conventional laminated structures [5,6,7]. During a single winding cycle, the wound fiber bundle intersects with the previous wound fiber bundle, resulting in an undulating architecture. The FWC structures usually have the appearance of diamond-shaped characteristic patterns, which are arranged periodically. The distribution of fiber bundles within each diamond-shaped pattern can be divided into two kinds of regions: (1) the laminated region, where fiber bundles in the +φ and −φ directions are laid parallel without undulation; and (2) the cross-fluctuation region, where fiber bundles in the +φ and −φ directions intersect at the boundary area.

In the laminated region, the loading behavior is similar to that of laminated plates. Similarly to simple [±φ] laminated plate structures, the laminated region of filament wound plates also exhibits nonlinear shear behavior. However, the presence of fiber bundle boundaries causes boundary effects, leading to changes in the mechanical behavior at the laminated region. In the cross-fluctuation region, the undulating configuration of the fiber bundle causes an uneven distribution of stresses in the fiber bundle, which may reduce the in-plane mechanical properties in the undulating area. Additionally, the appearance of resin-rich regions between the fiber bundle gaps also alters the stress distribution in this area. The “nonlinear shear effect and boundary effect” of the laminated region and the “local effect” of the cross-fluctuation region make the prediction of the mechanical behavior and damage evolution process of the filament wound plates’ structure very complex and difficult to elucidate.

In previous studies, the majority of scholars have ignored the fiber cross-fluctuation, opting to instead adopt netting theory and classical laminate theory [8,9,10] to treat filament wound plates’ structure as laminated or antisymmetric laminated structures. Based on damage mechanics, the failure criteria of composite materials and stiffness degradation models (including a direct stiffness reduction [11] and progressive failure model [12,13]) were used to simulate the failure of FWC plates [14,15,16,17]. However, the appearance of a cross-fluctuation region makes the stress distribution and failure mode very complex. It is difficult to capture the influence of a fiber bundle winding pattern on the overall mechanical behavior of the structure by using the traditional laminated structure model to analyze the FWC structure, which includes limitations such as an understanding of the influence of the fiber winding pattern on the mechanical characteristics of the structure.

In recent years, with the development of cross-scale numerical analysis techniques, some scholars have begun to pay attention to the analysis of the stress and strain distribution and damage behavior of filament wound structures by taking into account the cross-undulating morphology of fiber bundles [18,19,20,21,22,23,24,25]. Rousseau [18] investigated the influences of different wound patterns on the loading behavior of glass fiber wound tubes and found that the denser the wound patterns, which determine the degree of fiber fluctuation, the lower the axial tensile strength of the tubes. M. Torres Arellano [19] created glass fiber cross-layered flat samples for a tensile test. During the test, DIC was used to obtain the strain field, and this was compared with the simplified zoned two-dimensional shell element finite element model simulation. It was found that crack initiation and propagation occurred along the fiber’s cross-undulating region. The test results indicate that the samples exhibited significant nonlinear characteristics. However, the numerical analysis only considered the zonal characteristics of the fiber arrangement, and did not consider the fiber fluctuation’s morphology in the cross area. Henry [20] developed a homogenization analysis process which took into account the fiber cross-fluctuation region. Additionally, the compression failure behavior of the fiber wound cylinder was predicted. It was also proven that the fiber’s initial failure, initiated in the fiber cross-fluctuation region, and the fiber fluctuation characteristics had a significant influence on the analysis of the load behavior of the wound structure. HH Mian [21] studied the influence of a winding pattern on the distribution of the strain field in a composite wound pressure vessel and pointed out that the impact of a winding pattern should be investigated to develop a more realistic modeling and analysis method for pressure vessels. Zhang [22] studied the elastic behavior of triaxial braided composites. Based on the fiber volume ratio, sample thickness, and microscopic image analysis, a typical triaxial braided cell model was determined. The effective elastic constants of the composites were predicted using an analytical model and an axial tensile test. Shen [23] proposed a meso-scale model to study fiber cross-undulation, which was divided into circular undulation and spiral undulation. Based on the meso-structural model and classical lamination theory, the stiffness calculation method for fiber wound composites was established. Takuya [24] conducted finite element analysis on the axial compression of unidirectional carbon fiber-reinforced plastics and used a fiber fluctuation model to study the initiation and propagation of fiber kinks. It was found that the matrix yield diffused in the direction orthogonal to fiber and promoted the shear deformation of the whole model, and the compressive strength of fiber cross-fluctuation model decreased with the increase in the average fiber angle. Emad [25] developed a meso-scale three-dimensional finite element model to study the effects of different fiber bundle fluctuations on the mechanical and thermal properties of fiber wound composite pipes.

The morphological characteristics of fiber cross-undulation have a significant influence on the mechanical behavior of the FWC structures. Due to various kinds of winding angles, bundle thickness may be used, and the winding patterns and accompanying undulating architecture of the fiber bundles are also different. However, there is still a lack of studies which have reported on the influence of fiber bundles’ undulating architecture.

In this paper, the effect of fiber cross-undulation’s morphological characteristics on the mechanical behavior of a fiber wound composite plate under a unidirectional tensile load was studied by means of an experiment and a numerical simulation. In the experiment, composite plate samples containing a ±55° fiber winding form and laminates of the same size were prepared. During the tensile test, DIC equipment was used to monitor the changes in the surface displacement field and strain field during the entire loading process. In a numerical study, a mesoscopic 3D finite element model was constructed in ABAQUS by taking into account the fiber cross-undulation characteristics. Analyses of both the nonlinear shear behavior and the progressive failure were conducted. The difference in the mechanical behavior between the FWC plates and the laminated plates was discussed, and the mechanism of the fiber bundle undulation architecture’s influence on the loading behavior was discussed. Finally, a numerical analysis was carried out on FWC plates with variable thicknesses and variable winding angles, and the influence of the thickness and winding angle on the mechanical characteristics of the FWC plates was explored.

## 2. Materials and Methods

### 2.1. Materials and Sample Preparation

Two types of samples of FWC plates and standard laminated plates were prepared, respectively, among which the standard laminated samples were used to provide control experimental data (Table 1).

The two types of samples were made of T300 unidirectional carbon fiber/epoxy resin prepreg, which were manually cut, laid, and prepared by the autoclave (RG21, Xi’an Longde Technology LLC, Xi’an, China) forming process. Each group of samples consisted of two layers of prepreg. The thickness of a single prepreg layer was 0.2 mm, and the thickness of the sample was 0.4 ± 0.01 mm.

**Figure 1:**
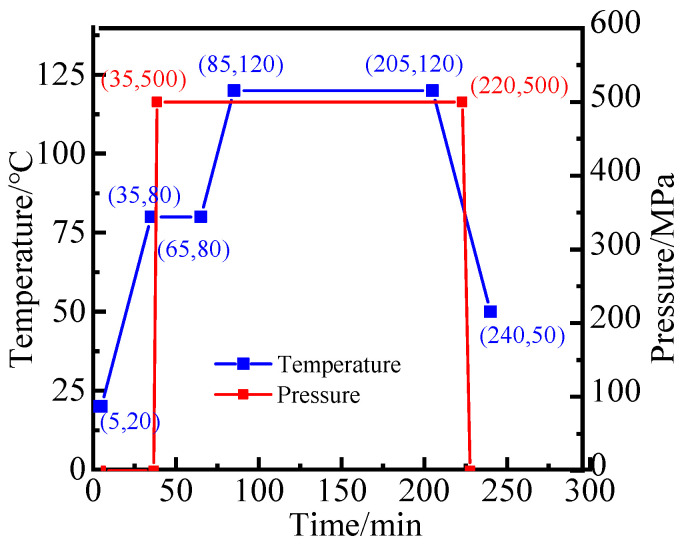
Curing temperature and pressure curves of T300/epoxy prepreg.

The preparation process of the sample of FWC plates was as follows: First, the prepreg fiber strip with a width of 6 mm was cut out; then, by referring to the fiber bundle cross-undulation pattern of the ±55° spiral winding layer, it was laid into an imitated winding structure plate containing the fiber bundle cross-undulation pattern. Then, the process curve, as shown in Figure 1, was used. After curing, the sample, with a length × width of 185 mm × 50 mm, was cut out, and the end surfaces were polished and pasted with a tab to avoid end slip and to ensure that the failure position was in the middle part of the sample.

**Figure 2:**
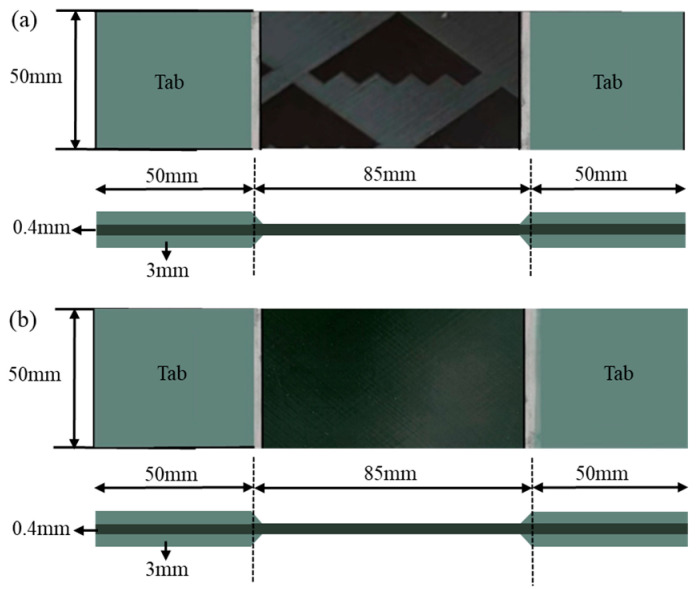
Schematic diagram of two composite samples ((**a**) FWC plate, (**b**) laminated plate).

The preparation process of the laminated plate sample was as follows: a rectangular prepreg of ±55° 300 mm × 600 mm was cut out and compacted after laying. The curing process was consistent with that of the FWC sample. After demolding, a sample with a length × width of 185 mm × 50 mm was also cut, and the end face was polished and pasted with a reinforcing piece. The dimensions of the two groups of samples are shown in Figure 2.

### 2.2. Testing and Characterization Methods

**Figure 3:**
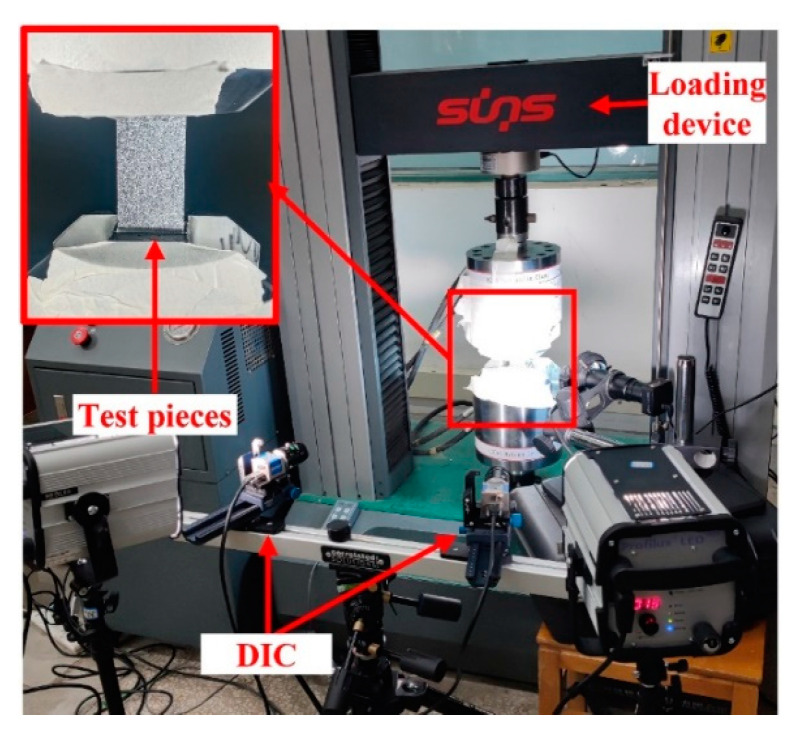
Schematic diagram of experimental test.

Two sets of samples were loaded under tension by an electronic universal testing machine, as shown in Figure 3. The test was controlled by displacement at a speed of 0.5 mm/min, and the real-time load–displacement curve was recorded during the test. Additionally, a 3D-DIC device was used to monitor the distributions of displacement and strain on the surfaces of the samples during loading.

## 3. Experimental Results and Discussion

### 3.1. Test Results

**Figure 4:**
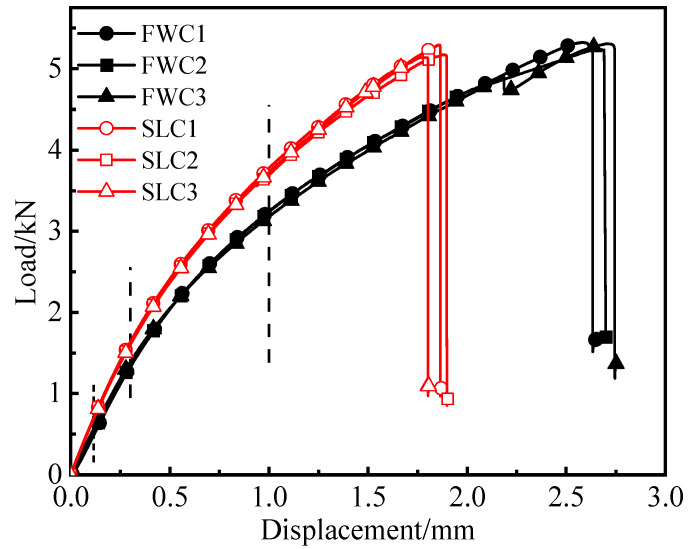
Experimental load–displacement curves (the dotted line: displacement at 0.1 mm,0.3 mm and 1.0 mm).

This section presents the load–displacement curves of the tensile tests of the FWC plates and laminated plates, as well as the distributions of the strain monitored by DIC. Figure 4 shows the load–displacement curves of the FWC plates and the laminated plates.

The displacement–load curves of the samples in each group showed good repeatability, with little dispersion within the groups. The load of the samples in each group presented nonlinear behavior along with the increase in the displacement, which was similar to the results reported in the literature [26,27,28].

By comparing the test results of the two groups of samples, it can be seen that the initial stiffness and later tangential stiffness of the FWC plates were lower than those of the laminated structure. The failure load of the FWC plates was similar to that of the laminated plates, both of which were 5.2 kN. The failure displacement of the FWC plates was significantly larger than those of the laminated plates, which were 2.7 mm and 1.8 mm, respectively.

FWC structures exhibited lower stiffness and greater failure displacement, which are both related to the deformation characteristics of the cross-undulation region. In the following chapters, the strain nephogram results of the FWC structure and laminated structure monitored by DIC during the test will be analyzed.

**Figure 5:**
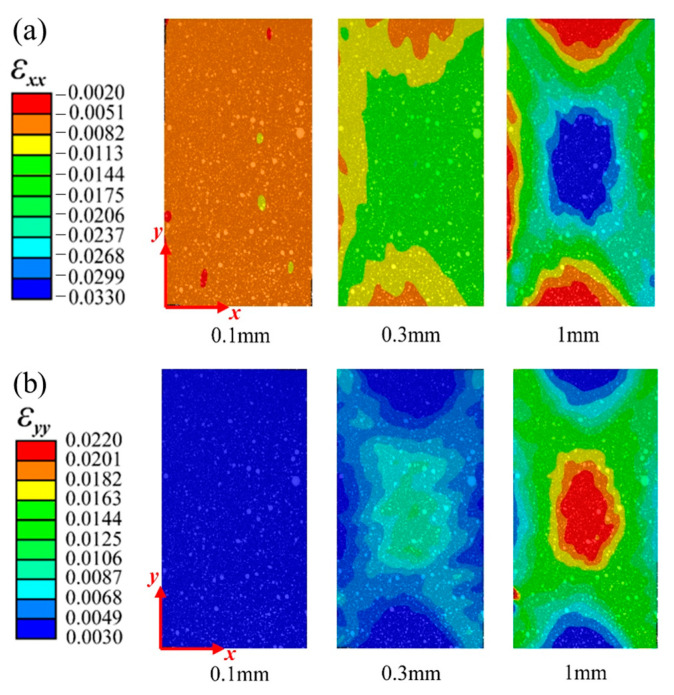
Variation in strain distributions during the tensile test of the laminated plates ((**a**) $\varepsilon_{\mathrm{xx}}$, (**b**) $\varepsilon_{\mathrm{yy}}$).

**Figure 6:**
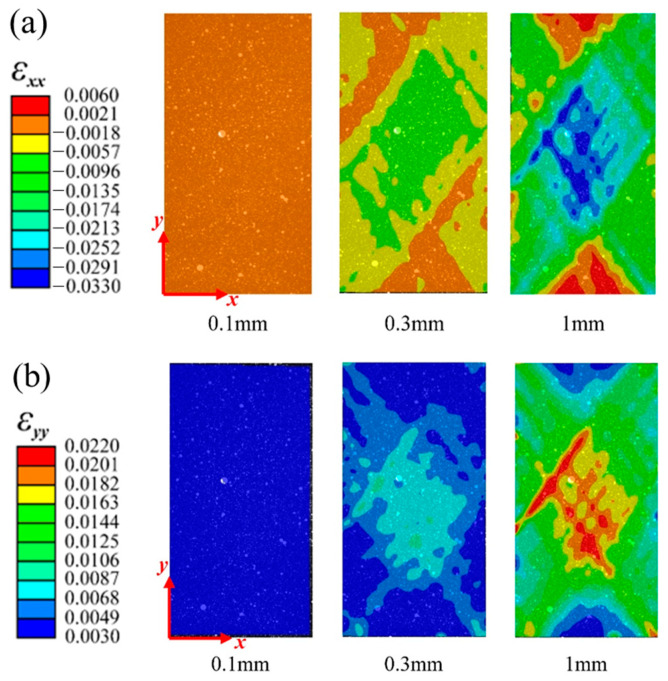
Variation in strain distributions during the tensile test of the FWC plates ((**a**) $\varepsilon_{\mathrm{xx}}$, (**b**) $\varepsilon_{\mathrm{yy}}$).

Figure 5 and Figure 6, respectively, show the variation in the horizontal strain ($\varepsilon_{\mathrm{xx}}$) and vertical strain ($\varepsilon_{\mathrm{yy}}$) nephograms of the laminated structure and FWC structure, which were monitored and analyzed by DIC under three different displacement loads (0.1 mm, 0.3 mm, and 1 mm, corresponding to the dotted line marks in Figure 4).

It can be seen from the figure that the strain concentration (including $\varepsilon_{\mathrm{xx}}$ and $\varepsilon_{\mathrm{yy}}$) occurred in the middle region of the sample in both the laminated structure group and the FWC structure group, which was mainly caused by the strong constraint on the clamping positions at both ends during the off-axial loading process, similar to the report in Refs. [29,30,31]; however, this is not the focus of this study. By comparing the two groups of samples, it was found that the strain concentration area of the laminated structure was relatively uniform, and the high strain area was elliptical and relatively small. In the FWC structure, the high strain interval was rhomboid, the area was relatively large, and the strain concentration was especially more significant in the local region of cross-undulation.

### 3.2. Analysis of Test Results

**Figure 7:**
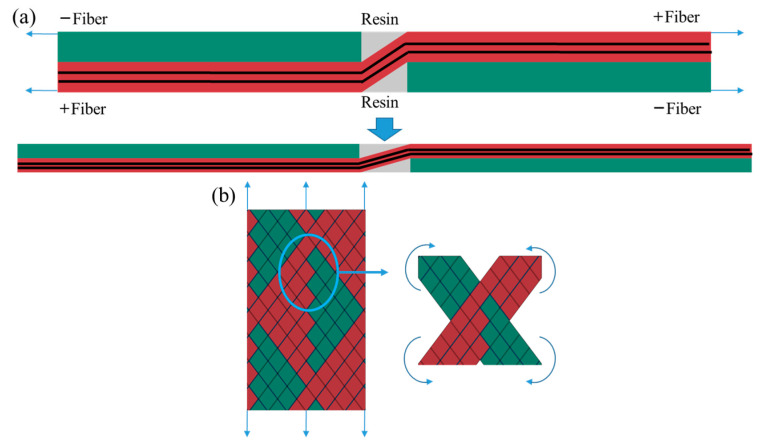
Schematic diagram of fiber stretching and deflection: (**a**) fiber stretching deformation, (**b**) fiber deflection (red: positive angle fiber, green: negative angle fiber, black: fiber direction).

The typical characteristics of the cross-fluctuation region include resin enrichment; the fiber fluctuates in the thickness direction and is deflected in the direction of the main load. Under tensile load, the ±55° fiber bundle stretched and was deflected simultaneously, as shown in Figure 7. Stretching is a unique deformation behavior of FWC plates. The +55° fiber’s bending angle decreased continuously, and the −55° fiber was extruded from the normal direction. At the same time, the resin-rich region was subjected to tensile and shear loads, resulting in a strain concentration in the cross-undulating region. However, there was only in-plane deflection caused by a fiber off-axis force in the laminated plates, and interlayer shear interaction generated ±55° layers. The deformation mechanism of FWC plates is much more complicated. In addition to the interlayer shear interaction, there was friction and extrusion between the ±55° fiber bands in the cross-undulating region. All of these led to a stress and strain concentration in the cross-fluctuation region. In the process of loading, the undulating fiber bundles are straightened gradually, and friction between fiber bundles may occur, leading to a decrease in the stiffness of the FWC plates and a relatively large failure displacement.

## 4. FEA Modeling

### 4.1. Finite Element Models

**Figure 8:**
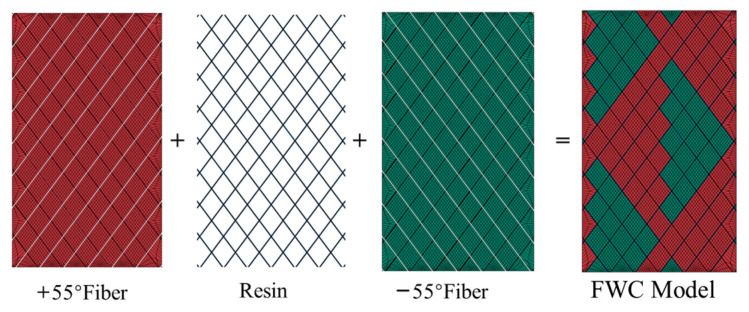
Finite element model assembly diagram.

The dimensions of the meso-scale finite element model of the FWC plate created in this paper were 50 mm × 85 mm × 0.4 mm, and the fiber bundles were successively combined in two winding directions of ±55°, as shown in Figure 8. There were two layers in the model, and resin was used to fill the cross-undulating gap in the fiber to form a resin-rich area. The +55° fiber band, −55° fiber band, and resin-rich region formed a complete winding model. A fixed constraint was applied to the bottom edge of the model, and a uniform displacement load was applied to the top edge of the model.

The finite element model of the laminated plate was composed of only two layers of solid elements, which were assigned material orientations of +55° and −55°, respectively. The model size was the same as the FWC plate.

Both the laminated plate model and the FWC plate model took into account the nonlinear shear behavior and progressive failure of the materials, as detailed in Section 3.2. Most of the properties of unidirectional laminates were obtained by experimental tests following the corresponding ASTM standards [32,33,34,35]. Poisson’s ratio was measured by strain gages during the tensile tests. The material properties obtained from the experimental results are marked with experimental errors in Table 2. The rest of the parameters were derived from Ref. [36].

### 4.2. Nonlinear Shear Behavior and Progressive Failure Analysis Methods

The carbon fiber-reinforced polymer exhibits nonlinear shear behavior when the composite is subjected to off-axial tensile load [37,38]. In this study, the numerical analysis employed the Ramberg–Osgood equation [39,40,41] in the mechanical constitutive model of the single-layer plate to describe the nonlinear shear behavior. The shear stress–strain relationship could be expressed as follows:

(1) $$\tau =\frac{G_{0}\gamma }{{\left[1+{\left(G_{0}\gamma /\tau_{0}\right)}^{n}\right]}^{1/n}}$$

where τ represents shear stress, γ represents shear strain, $G_{0}$ represents the initial shear modulus, $\tau_{0}$ represents the asymptotic value of τ when γ approaches infinity, and *n* represents the shape parameter.

Based on the above equation, the nonlinear shear stiffness *G* of the material could be defined, and its nonlinear relationship with shear strain was given by:

(2) $$G=\frac{d\tau }{d\gamma }=\frac{G_{0}}{{\left[1+{\left(G_{0}\gamma /S\right)}^{n}\right]}^{1/n}}$$

The implementation of the above nonlinear shear constitutive equation was conducted using the user subroutine VUMAT in the Abaqus finite element platform. The material parameters *n* and $\tau_{0}$ of the experimental material system were determined by fitting the shear experiments of standard ±45° laminates (ASTM D3518). In this paper, the value of *n* for the Ramberg–Osgood equation of the material system was 2.4, with S at 135 MPa.

The damage initiation and evolution of composite materials were described using the progressive failure model. The Hashin failure [42,43,44] criterion was used to predict the initiation of the failure of unidirectional fiber bundles, with the following formulas:

Fiber tensile failure ($\sigma_{11}>0$):

(3) $${\left(\frac{\sigma_{11}}{X_{T}}\right)}^{2}+{\left(\frac{\tau_{12}}{X_{T}}\right)}^{2}+{\left(\frac{\tau_{13}}{S_{13}}\right)}^{2}\geq 1$$

Fiber compressive failure ($\sigma_{11}<0$):

(4) $${\left(\frac{\sigma_{11}}{X_{C}}\right)}^{2}\geq 1$$

Matrix tensile failure ($\sigma_{22}>0$):

(5) $${\left(\frac{\sigma_{22}}{Y_{T}}\right)}^{2}+{\left(\frac{\tau_{12}}{S_{12}}\right)}^{2}+{\left(\frac{\tau_{23}}{S_{23}}\right)}^{2}\geq 1$$

Matrix compressive failure ($\sigma_{22}<0$):

(6) $${\left(\frac{\sigma_{22}}{Y_{C}}\right)}^{2}+{\left(\frac{\tau_{12}}{S_{12}}\right)}^{2}+{\left(\frac{\tau_{23}}{S_{23}}\right)}^{2}\geq 1$$

In this context, $\sigma_{11}$ and $\sigma_{22}$ represent normal stresses in the fiber direction and perpendicular to the fiber direction, respectively. $\tau_{12}$, $\tau_{13}$, and $\tau_{23}$ are the shear stresses, and $X_{T}$ and $X_{C}$ are the tensile and compressive strengths in the fiber direction, respectively.

Following initial damage, the composite material damage extended with the increasing load, and when the corresponding critical strain energy release rate (fracture energy) was reached, the composite material completely failed. For the four failure modes, namely, fiber tension, fiber compression, matrix tension, and matrix compression, the damage state variables were defined as follows:

(7) $$d_{11}^{t}\left(\varepsilon_{11}\right)=\frac{\varepsilon_{f,1}^{t}}{\varepsilon_{f,1}^{t}-\varepsilon_{0,1}^{t}}\left(1-\frac{\varepsilon_{0,1}^{t}}{\varepsilon_{11}}\right)$$

(8) $$d_{11}^{c}\left(\varepsilon_{11}\right)=\varepsilon_{f,1}^{c}\left(\varepsilon_{f,1}^{c}-\varepsilon_{0,1}^{c}\right)\left(1+\frac{\varepsilon_{0,1}^{c}}{\varepsilon_{11}}\right)$$

(9) $$d_{22}^{t}\left(\varepsilon_{22}\right)=\frac{\varepsilon_{f,2}^{t}}{\varepsilon_{f,2}^{t}-\varepsilon_{0,2}^{t}}\left(1-\frac{\varepsilon_{0,2}^{t}}{\varepsilon_{22}}\right)$$

(10) $$d_{22}^{c}\left(\varepsilon_{22}\right)=\varepsilon_{f,2}^{c}\left(\varepsilon_{f,2}^{c}-\varepsilon_{0,2}^{c}\right)\left(1+\frac{\varepsilon_{0,2}^{c}}{\varepsilon_{22}}\right)$$

In this equation, $\varepsilon_{0,1}^{t}=\frac{X_{t}}{C_{11}}$, $\varepsilon_{0,1}^{c}=\frac{X_{c}}{C_{11}}$, $\varepsilon_{0,2}^{t}=\frac{Y_{t}}{C_{22}}$, $\varepsilon_{0,2}^{c}=\frac{Y_{c}}{C_{22}}$, $C_{11}$ and $C_{22}$ denote the stiffness matrices and $\varepsilon_{f,i}^{t}$ and $\varepsilon_{f,i}^{c}$ represent the element failure strains. The relationship between the strain energy release rate’s critical value and the failure strains can be described as $\varepsilon_{f,i}^{t}=\frac{2G_{\mathrm{ic}}^{t}}{\sigma_{t}l}$, $\varepsilon_{f,i}^{c}=\frac{2G_{\mathrm{ic}}^{c}}{\sigma_{c}l}$, where *l* is the characteristic length of the element. In this paper, the characteristic length could be approximately solved using the embedded function charLength with VUMAT in ABAQUS [45].

### 4.3. Numerical Results and Discussion

**Figure 9:**
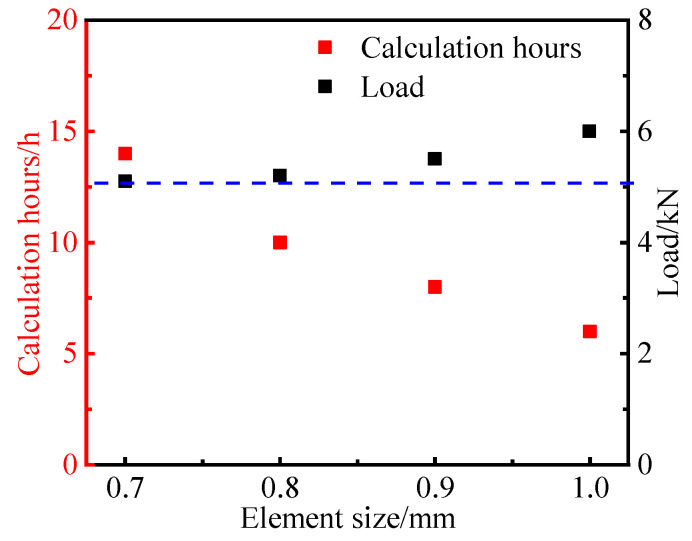
The effect of element size on the simulation results (the blue dashed line shows the load is becoming stable).

The effects of the element size on the simulation results were studied in order to ensure that an appropriate element size was used. All models were calculated using 10 central processing units. The element selected in the finite element model was the C3D8R solid element. As seen in Figure 9, as the element size reduced to 0.8 mm, the tensile force began to stabilize. However, the computational time increased dramatically as the element size decreased. Therefore, the mesh size at the edge of the hole was set at 0.8 mm in this study to strike a balance between the computational efficiency and computational accuracy.

**Figure 10:**
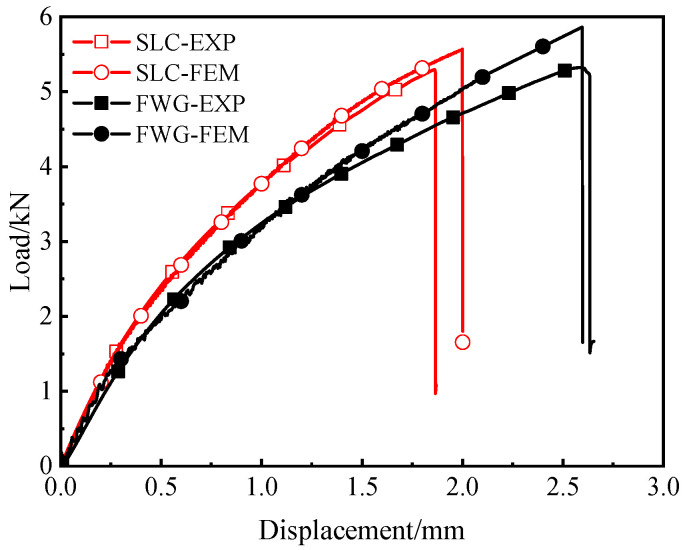
Comparison of load–displacement curves between finite element analysis and experimental test.

Figure 10 shows the load–displacement curves of the laminates and the FWC structure obtained by the numerical simulation; corresponding experimental results are also provided for a comparative analysis. As can be seen from the figure, the load–displacement curves obtained by simulation were generally in good agreement with the experimental results, regardless of the laminated plates or the FWC plates.

The simulated curves of both the laminated plates and the FWC plates showed significant nonlinear behavior, which was consistent with the experimental results. The results showed that it was effective to introduce the nonlinear shear stress–strain deformation feature into the material constitutive by means of user subroutine (VUMAT) in this numerical analysis.

The stiffness of the load–displacement curve predicted by the numerical model of the laminated plates was basically the same as that of the experiment, and was obviously higher than that of the FWC plates.

**Figure 11:**
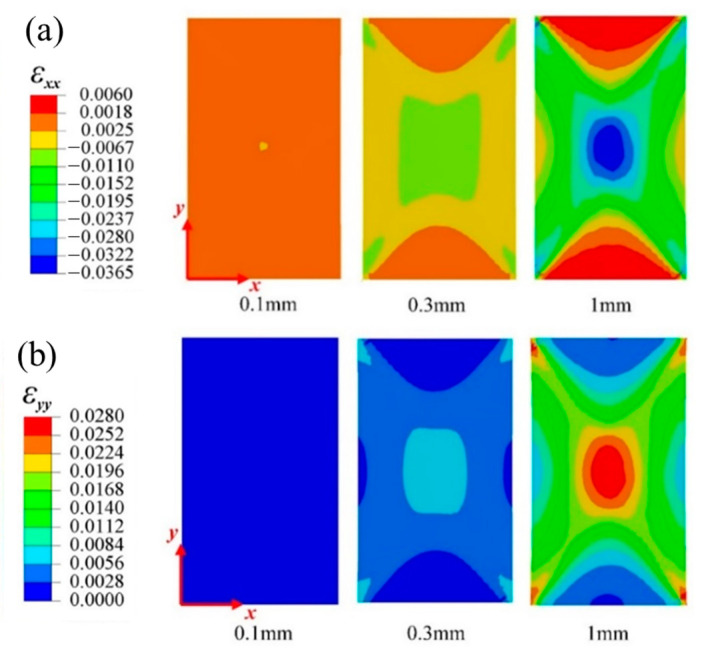
Variation in the strain distributions predicted by FEA for a laminated plate ((**a**) $\varepsilon_{\mathrm{xx}}$, (**b**) $\varepsilon_{\mathrm{yy}}$).

**Figure 12:**
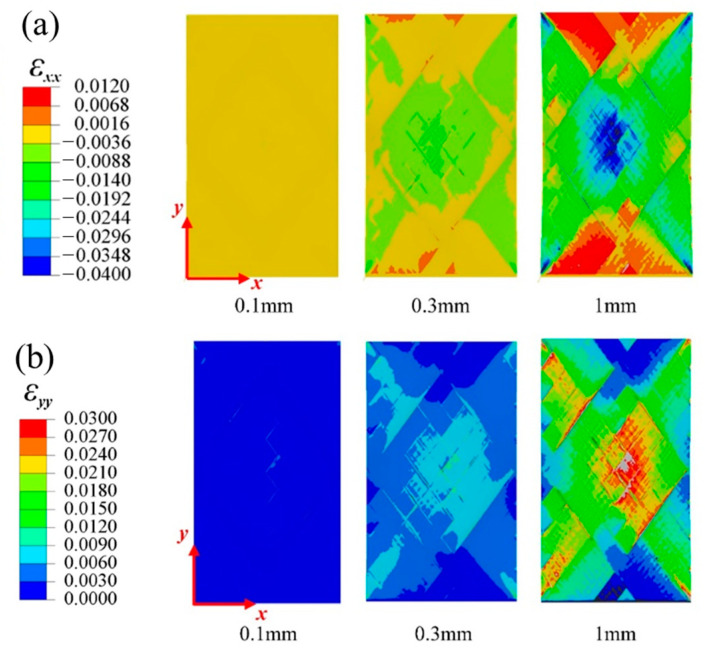
Variation in the strain distributions predicted by FEA for an FWC plate ((**a**) $\varepsilon_{\mathrm{xx}}$, (**b**) $\varepsilon_{\mathrm{yy}}$).

Figure 11 and Figure 12 show the strain field nephogram, obtained through numerical prediction, for the laminated and FWC plates, respectively. The selected time frames are consistent with those of the experimental results, and correspond to loading displacements of 0.1 mm, 0.3 mm, and 1 mm, respectively.

Regarding the aspect of the strain distribution, the numerical analysis results were consistent with the experiment. As the loading progressed, the fiber crossover fluctuation region in the middle of the diamond-shaped characteristic unit in the sample had a larger strain distribution in both the horizontal and vertical directions than the surrounding laminated region. At a loading displacement of 1 mm, there was an obvious strain concentration phenomenon in the fiber crossover fluctuation area of the diamond-shaped characteristic unit and at the boundary of the diamond-shaped unit.

### 4.4. Progressive Damage Process

**Figure 13:**
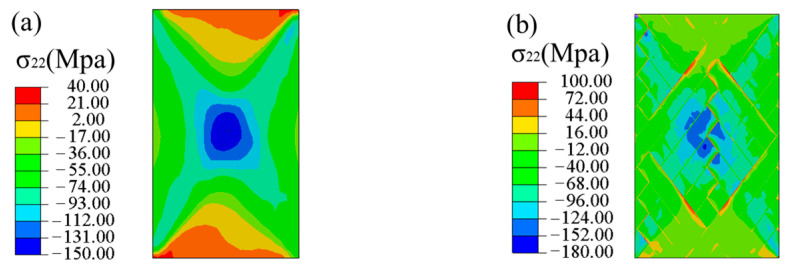
Transverse stress nephograms, predicted by FEA ((**a**) laminated plate, (**b**) FWC plate).

Figure 13 shows the numerical predicted stress ($\sigma_{22}$) nephograms of the laminated and FWC plates under a tension displacement of 1 mm. From the figure, it can be seen that the laminates were compressed in the transverse direction in most areas, except in the gripper end. Except for the upper and lower ends of the winding structure, the bundles were compressed in the transverse direction in most areas of the plates. Small areas in the cross-fluctuation region of the fiber and at the edge of the rhomboid characteristic element were stretched in transverse direction, and significant stress concentration were observed. A part of the cross-fluctuation region was subjected to the extrusion caused by bundle shear, so there was a phenomenon of stress concentration in the transverse direction.

**Figure 14:**
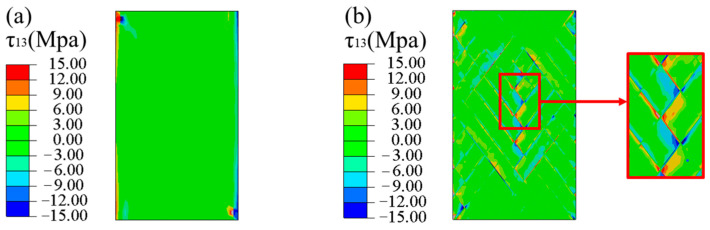
Shear stress $\tau_{13}$ nephograms, predicted by FEA ((**a**) laminated plate, (**b**) FWC plate).

Figure 14 shows the stress ($\tau_{13}$) nephograms of the laminated and FWC plates under a tension displacement of 1 mm. Except for a few areas of stress concentration at the gripper end of the laminated plate, the shear stress in most areas was relatively uniform, while the FWC plate had an obvious stress concentration phenomenon in the fiber cross-fluctuation region.

**Figure 15:**
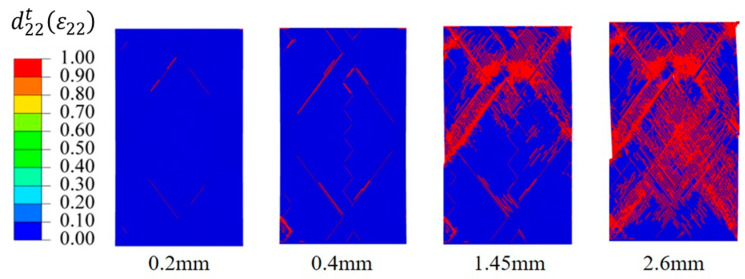
Progressive damage progress of matrix tensile damage to the FWC plate.

Figure 15 shows the progressive failure process of the FWC structure. Under the axial tensile loading, there was no fiber tensile or compression failure, and the main failure mode was matrix failure under the tensile condition. When the loading displacement reached 0.2 mm, matrix damage began to appear at the edge of the diamond-shaped characteristic unit of the FWC plate, and a further analysis showed that the matrix failure was mainly dominated by shear stress $\tau_{12}$ and $\tau_{13}$. As the loading progressed, at a displacement of 0.4 mm, the matrix tensile damage at the center and the edge of the diamond-shaped characteristic unit and the crossover fluctuation area was further extended. When the loading displacement reached 1.45 mm, the matrix on the upper crossover edge of the diamond pattern underwent matrix tensile/shear damage. At a displacement of 2.6 mm, macroscopic shear deformation occurred between the fiber bundles; the test results were consistent, indicating that the sample lost its bearing capacity.

## 5. Numerical Exploration on the Influence of Bundle Thickness and Winding Angle

In the context of FWC structures, variations in the helix winding angle and fiber thickness give rise to different fiber winding patterns. In order to investigate the effects of the winding angle and fiber bundle thickness on the FWC plate, a comparative analysis was conducted on ±55° FWC plates with altered fiber layer thicknesses based on the modeling and numerical analysis methods proposed in Section 3. Additionally, ±15°, ±25°, and ±45° FWC plate models were established and compared with the same-sized laminated plates to explore the effects of different winding angles on the stiffness degradation of the FWC plate.

### 5.1. Influence of Bundle Thickness

**Figure 16:**
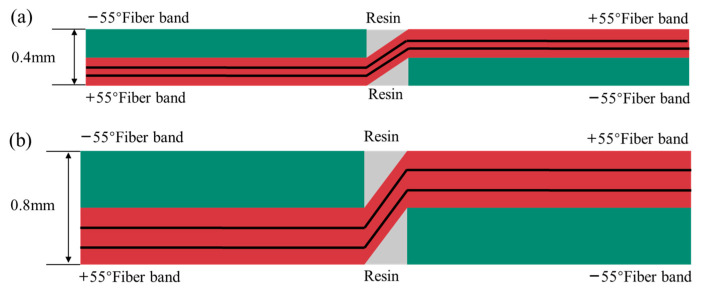
Sectional diagram of FWC structures with variable thicknesses ((**a**) 0.4 mm, (**b**) 0.8 mm).

On the basis of the numerical analysis of the ±55° FWC plates presented in Section 3, we doubled the thickness of a single layer to simulate the stress characteristics of fiber winding layers of different thicknesses during the winding process. The cross-sectional schematic diagram is shown in Figure 16.

**Figure 17:**
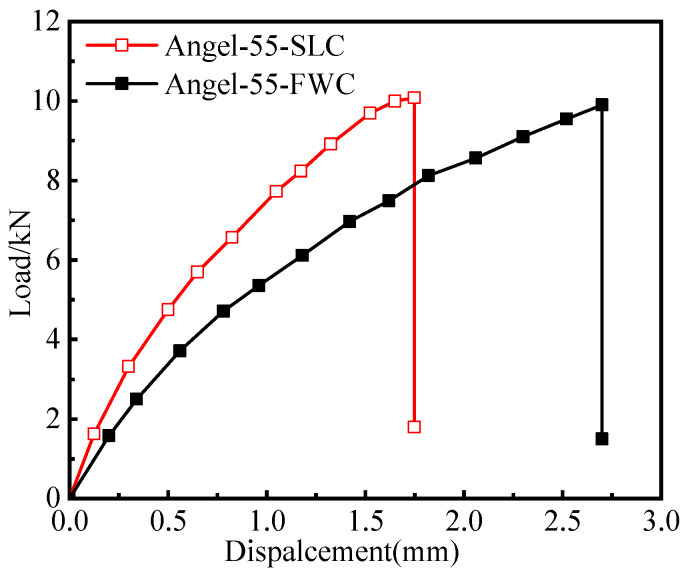
Load-displacement curve of the ±55° FWC plate and the laminated plate with a thickness of 0.8 mm.

After conducting numerical simulation calculations, the load–displacement curves of the ±55° FWC plate with a thickness of 0.8 mm were obtained, as shown in Figure 17. In comparison to the load–displacement curve of the ±55° FWC plate with a thickness of 0.4 mm, which was presented earlier, it was observed that the stiffness reduction effect of the 0.8 mm FWC plate was more obvious than that of the 0.4 mm FWC plate, and it was even more distinct relative to the same-sized laminated plate.

**Figure 18:**
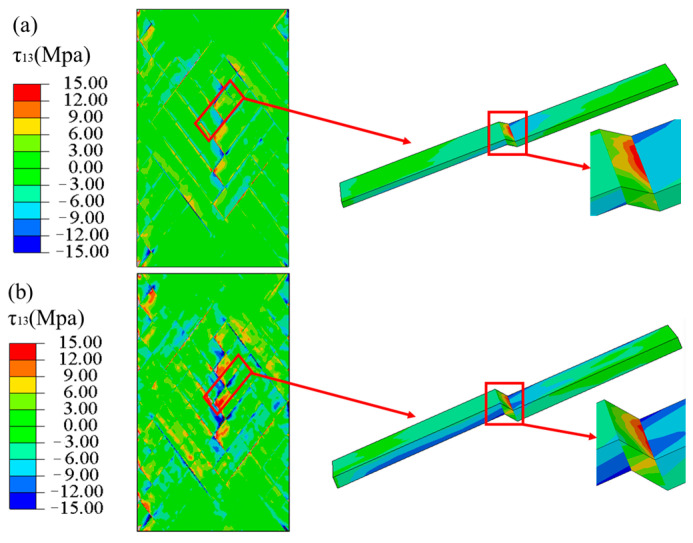
The stress nephogram of $\tau_{13}$ in the corrugated regions of FWC plates with different thicknesses ((**a**) 0.4 mm, (**b**) 0.8 mm).

The stress nephogram for the undulating region of the FWC plates with two different thicknesses is shown in Figure 18. The stress concentration in the undulating areas is more pronounced in the 0.8 mm thickness FWC plate, and the fiber bending effect is more obvious.

### 5.2. Influence of Winding Angle

**Figure 19:**
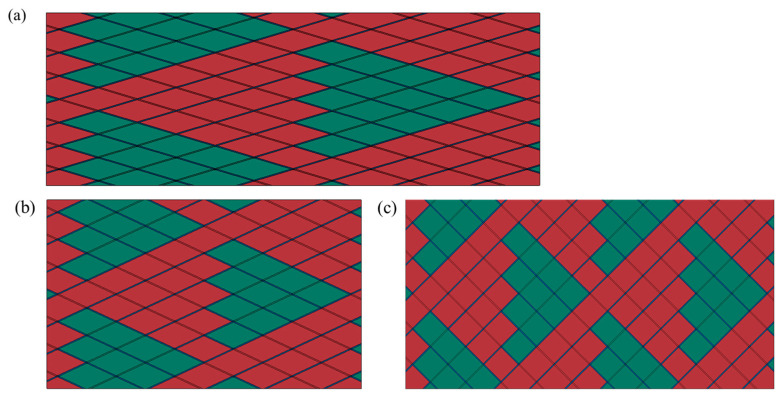
Schematic of the FWC models with different winding angles ((**a**) ±15°, (**b**) ±25°, (**c**) ±45°) (red: positive angle fiber, green: negative angle fiber).

In the FWC structure, it is common to use different angles for winding. In order to study the influence of the winding angle on the decrease in the stiffness of the FWC plates, we created models of ±15°, ±25°, and ±45° FWC plates, with sizes of 165 mm × 48 mm × 0.4 mm, 80 mm × 48 mm × 0.4 mm, and 92 mm × 48 mm × 0.4 mm, respectively, as shown in Figure 19. The utilized numerical analysis method was the same as that in the previous section.

**Figure 20:**
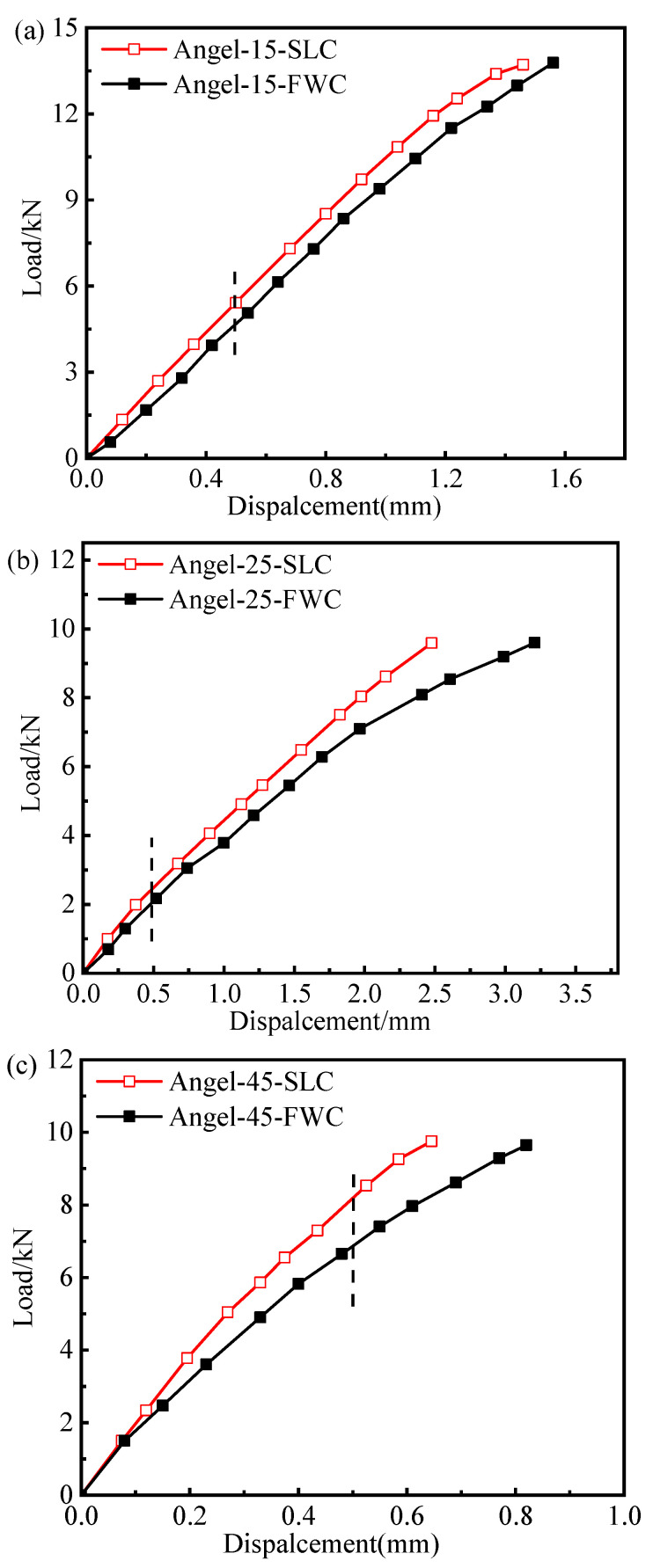
The load–displacement curves for the laminated and FWC plates with different winding angles ((**a**) ±15°, (**b**) ±25°, (**c**) ±45°) (the dashed line: displacement at 0.5 mm).

**Figure 21:**
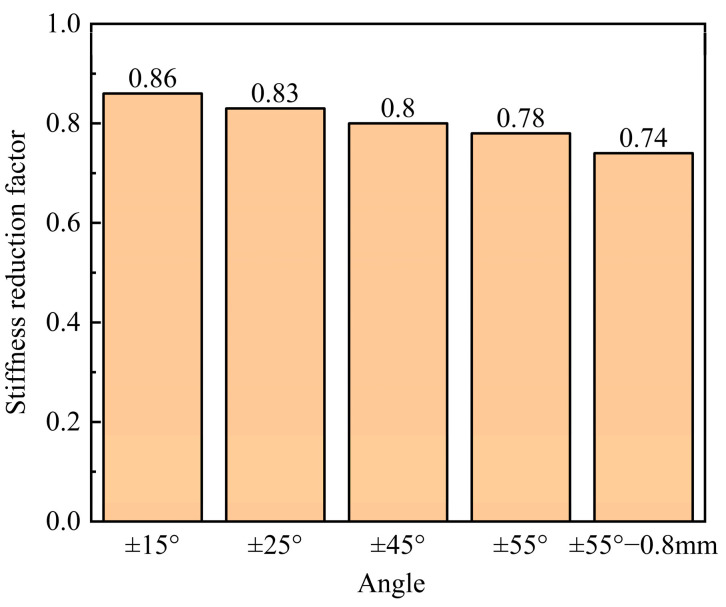
Stiffness reduction coefficient prediction of FWC plates with different winding angles.

Figure 20 shows the ±15°, ±25°, and ±45° load–displacement curves of the FWC plates, as well as the laminated plates of the same size, obtained by numerical simulation. The stiffness when the loading displacement reached 1% of the length in the tensile direction was captured and compared with the stiffness values of laminates of the corresponding size. As shown in Figure 21,the smaller the winding angle was, the weaker the stiffness reduction effect became.

**Figure 22:**
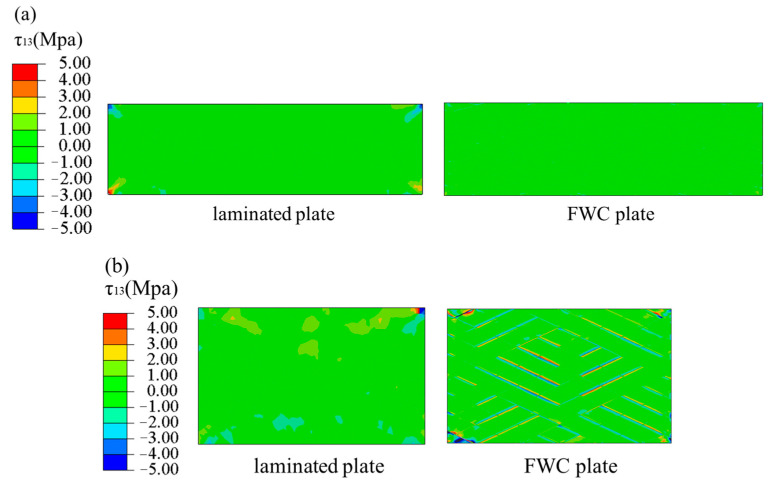

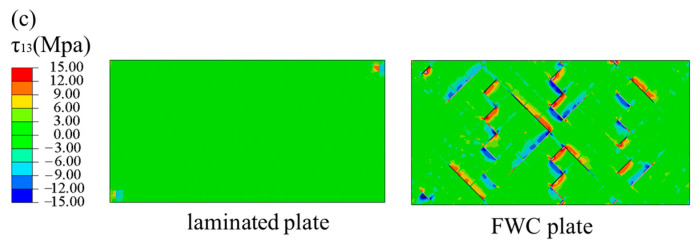
Stress nephogram $\tau_{13}$ of laminated and FWC plates with different angles ((**a**) ±15°, (**b**) ±25°, (**c**) ±45°).

The stress nephogram $\tau_{13}$, in Figure 20, of the three angles of the FWC plates at the dotted line, where the tensile length was 0.5 mm, was extracted, and it is shown in Figure 22. For the FWC plate with a winding angle of ±15°, there was almost no stress concentration phenomenon in the winding undulation region, similar to the results of the laminated plate. For the FWC plate with a winding angle of ±25°, there was a slight stress concentration phenomenon in the winding undulation region, differing from the results of the laminated plate. For the FWC plate with a winding angle of ±45°, there was an obvious stress concentration phenomenon in the winding undulation region, greatly differing from the results of the laminated plate. It was found that the more serious the stress concentration, the lower the stiffness reduction coefficient.

## 6. Conclusions

The loading behaviors of the FWC plates and laminated plates were analyzed by means of experimental and numerical simulation methods. The influence of the fiber bundles’ undulating architecture on the tensile behavior of filament wound composite laminates was studied.

Two layers of carbon fiber/epoxy composite plate specimens, both laminated and FWC and with an angle of ±55°, were made, and the tensile test results showed that the stiffness of the laminated plate was significantly greater than that of the FWC plate. An obvious strain concentration in the middle of the fiber cross-fluctuation region was observed in the FWC plate. The strain concentration in the FWC plates was mainly caused by the resin enrichment and fiber waving in the cross-fluctuation region.

A numerical analysis method was proposed to predict the failure process of the laminated plates and the FWC plates. The load–displacement curves obtained from the numerical analysis were consistent with the experimental results. It was demonstrated that the meso-scale model created in this study can capture the influence of fiber bundle cross-undulation morphology, as the predicted stiffness, strain distribution, and failure mode were consistent with the test results. The resin enrichment and straightening effect caused by fiber cross-undulation led to a reduction in the overall stiffness of the FWC plates.

Further numerical studies showed that the stiffness reduction coefficient of filament wound plates with winding angles of ±55° decreased from 0.78 to 0.74 as the bundle thickness increased from 0.4 mm to 0.8 mm. The stiffness reduction coefficients of filament wound plates with wound angles of ±15°, ±25°, and ±45° were 0.86, 0.83, and 0.8, respectively.

## Figures and Tables

**Table 1:** Label, number and description of samples.

Label	Number of Sample	Description
FWC	3	Filament wound plates
SLC	3	Laminated plates

**Table 2:** Material properties of composite laminates and resin.

Parameter	Value	Parameter	Value	Parameter	Value
*E*_11_	127 ± 3 GPa	*E*_22_	7.9 ± 0.3 GPa	*E*_33_	7.9 ± 0.3 GPa
*v*_12_	0.35 ± 0.01	*v*_13_	0.35 ± 0.01	*v*_23_	0.45 ± 0.01
*G*_12_	2.1 ± 0.2 GPa	*G*_13_	2.1 ± 0.2 GPa	*G*_23_	4.8 GPa
*X*_T_	2.0 ± 0.08 GPa	*X*_C_	1.2 ± 0.02 GPa	*Y*_T_	38.5 ± 3 MPa
*Y*_C_	180.7 ± 5 MPa	S	135.0 ± 3 MPa	*G*_ft_	133 N/mm [36]
*G*_fc_	60 N/mm [36]	*G*_mt_	0.352 ± 0.03 N/mm	*G*_mt_	1.45 N/mm [36]
*E*	3.0 GPa	*μ*	0.37		

Note: *E*—elastic modulus, *v*—Poisson’s ratio, *G*—shear modulus, 1—the longitudinal direction, 2—the transverse direction, 3—the thickness direction of the layer, *X*_T_—longitudinal tensile strength, *X*_C_—longitudinal compressive strength, *Y*_T_—transverse tensile strength, *Y*_C_—transverse compressive strength, *S*—in-plane shear strength, *G*it and *G*ic—critical values of strain energy release rate.

## Data Availability

The data presented in this study are available on request from the corresponding author.
